# Alpha-Ketoglutarate as a Molecule with Pleiotropic Activity: Well-Known and Novel Possibilities of Therapeutic Use

**DOI:** 10.1007/s00005-016-0406-x

**Published:** 2016-06-20

**Authors:** Barbara Zdzisińska, Aleksandra Żurek, Martyna Kandefer-Szerszeń

**Affiliations:** Department of Virology and Immunology, Institute of Microbiology and Biotechnology, Maria Curie-Sklodowska University, Akademicka 19, 20-033 Lublin, Poland

**Keywords:** Alpha-ketoglutarate, Antioxidative factor, Dietary supplement, Immunomodulatory agent, Bone anabolic agent, Anticancer agent

## Abstract

Alpha-ketoglutarate (AKG), an endogenous intermediary metabolite in the Krebs cycle, is a molecule involved in multiple metabolic and cellular pathways. It functions as an energy donor, a precursor in the amino acid biosynthesis, a signalling molecule, as well as a regulator of epigenetic processes and cellular signalling via protein binding. AKG is an obligatory co-substrate for 2-oxoglutarate-dependent dioxygenases, which catalyse hydroxylation reactions on various types of substrates. It regulates the activity of prolyl-4 hydroxylase, which controls the biosynthesis of collagen, a component of bone tissue. AKG also affects the functioning of prolyl hydroxylases, which, in turn, influences the function of the hypoxia-inducible factor, an important transcription factor in cancer development and progression. Additionally, it affects the functioning of enzymes that influence epigenetic modifications of chromatin: ten–eleven translocation hydroxylases involved in DNA demethylation and the Jumonji C domain containing lysine demethylases, which are the major histone demethylases. Thus, it regulates gene expression. The metabolic and extrametabolic function of AKG in cells and the organism open many different fields for therapeutic interventions for treatment of diseases. This review presents the results of studies conducted with the use of AKG in states of protein deficiency and oxidative stress conditions. It also discusses current knowledge about AKG as an immunomodulatory agent and a bone anabolic factor. Additionally, the regulatory role of AKG and its structural analogues in carcinogenesis as well as the results of studies of AKG as an anticancer agent are discussed.

## Introduction

Metabolites, small-molecular weight molecules, are the substrates and products of enzymatic reactions that occur naturally within cells. Among these substances, there is alpha-ketoglutarate (AKG; also known as 2-oxoglutarate, 2-oxopentanedioic acid), an endogenous intermediary metabolite in the Krebs cycle (the citric acid cycle or the tricarboxylic acid cycle, TCA) (Krebs and Johnson 1980). For many years, AKG has been an object of interest for researchers from various fields of science due to its essential role in several biological processes and its broad application scope.

AKG is an important biological molecule that plays a key role in multiple metabolic and cellular pathways. As a Krebs cycle metabolite, it regulates anabolic and catabolic TCA products and substrates, thereby regulating amino acid synthesis, ATP production, and reducing equivalent (NAD^+^/NADH) generation, which in turn can influence reactive oxygen species (ROS) levels (Krebs and Johnson 1980). AKG is also an obligatory co-substrate for 2-oxoglutarate-dependent dioxygenases (2-OGDDs) (McDonough et al. 2010; Schofield and Zhang 1999), a large group of phylogenetically conserved enzymes, which catalyse hydroxylation reactions on various types of substrates including proteins, nucleic acids, lipids, and metabolic intermediates. These enzymes require the presence of Fe(II) as a cofactor as well as O_2_ and AKG as co-substrates. In the hydroxylation reaction of the substrate, one oxygen atom from O_2_ is attached to a hydroxyl group in the substrate while the other one is taken up by AKG, which leads to decarboxylation of AKG and subsequent formation of CO_2_ and succinate. Ascorbic acid (vitamin C) also takes part in these reactions by inducing reduction of oxidised Fe(IV) to Fe(II) and restoring the activity of 2-OGDD enzymes. In humans, there are more than 60 different 2-OGDDs, and some of these enzymes play a key role in physiologically important processes such as the hypoxic response, fatty acid metabolism, nucleic acid repair and modification, and epigenetic regulation (Hausinger 2004; Rose et al. 2011). As a substrate of hydroxylases, belonging to OGDDs, AKG exerts an impact on prolyl/aspartyl/lysyl hydroxylations, which in turn regulates the stability of the hypoxia-inducible factor (HIF)-1 and collagen synthesis. Prolyl hydroxylases PHD1-3 influence the function of HIF-1 (Bruick and McKnight 2001; Epstein et al. 2001; Hirsilä et al. 2003), an important transcription factor in cancer development and progression, while prolyl-3 and prolyl-4 hydroxylases (P3H, P4H) control the biosynthesis of collagen (Kivirikko and Pihlajaniemi 1998), a very important component of bone tissue. AKG is also a required substrate of the Jumonji C domain containing lysine demethylases (KDM2-7), which are the major histone demethylases (Tsukada et al. 2006) and ten–eleven translocation hydroxylases (TET1-3) involved in DNA demethylation, which catalyse the oxidative decarboxylation of AKG, generating 5-hydroxy-methylcytosine (5-hmC) and leading to epigenetic effects (Ito et al. 2010; Tahiliani et al. 2009). Moreover, AKG binds and regulates G protein function, because it is a ligand for the G protein-coupled receptor (GPR99/GPR80 or OXGR1), which acts exclusively through a Gq/_11_-mediated pathway (He et al. 2004). Signalling through this pathway mobilises intracellular Ca^2+^ (via activation of phospholipase C), which acts as a diffusible second messenger regulating a wide range of vital cell functions, including cellular metabolism and growth as well as cell division and differentiation (Mizuno and Itoh 2009). In this way, AKG can also function as a signalling molecule. The GPR99 receptor has so far been found in kidney, placenta, testis, smooth muscles, trachea, and mast cells (He et al. 2004; Wittenberger et al. 2002), however, its physiological role has been recently described only in kidney, where it regulated the acid–base balance in the kidney tubules in an AKG-dependent manner (Tokonami et al. 2013).

Recently, it has been shown that supplementation of AKG to adult *Caenorhabditis elegans* delays ageing of this nematode. These studies revealed a novel binding protein of AKG, namely the ATP synthase beta subunit (Chin et al. 2014). This finding suggests that regulatory networks acted upon by AKG are more complex than it was previously supposed.

**Fig. 1:**
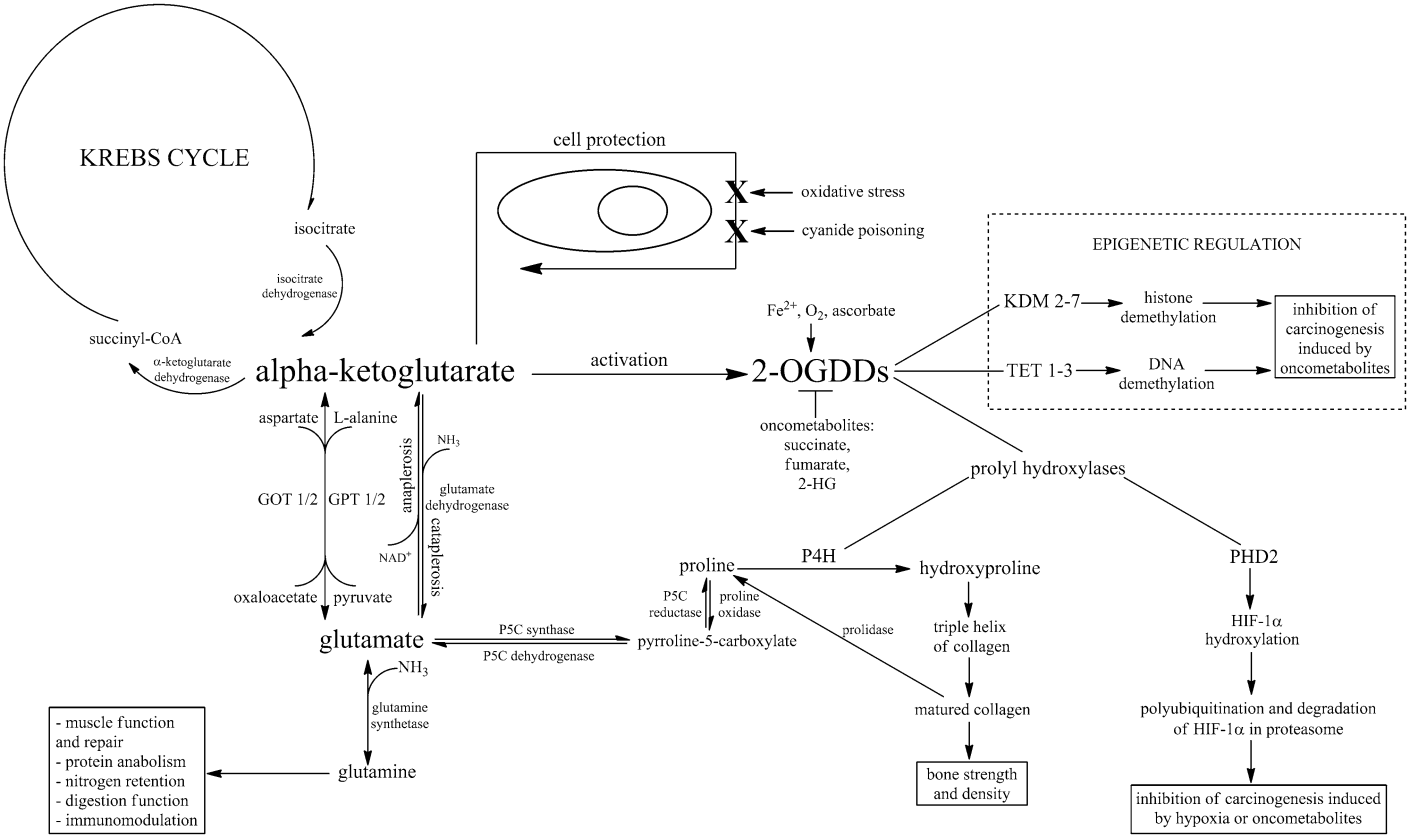
Schematic representation of the pleiotropic activity of the AKG molecule. Alpha-ketoglutarate is a precursor of glutamine which contributes to muscle repair, prevents protein catabolism, improves nitrogen retention, functions as an immunomodulatory molecule, and takes part in proper function of intestines. AKG is also involved in cell protection against oxidative stress and cyanide poisoning. It can also influence bone strength and density and inhibit carcinogenesis induced by oncometabolites or hypoxia by activating enzymes from the 2-OGDD family (2-oxoglutarate-dependent dioxygenases). Their action involves epigenetic regulation such as histone and DNA demethylation carried out by KDM 2–7 (Jumonji C domain containing lysine demethylases) and TET 1–3 (10–11 translocation hydroxylases), respectively, and non-epigenetic regulation, which includes activation of prolyl hydroxylases: P4H (prolyl 4-hydroxylase) involved in type I collagen biosynthesis and PHD2 (prolyl hydroxylase domain-containing protein 2) responsible for hydroxylation and thus inactivation of HIF-1α (hypoxia-inducible factor). Other cofactors for the 2-OGDD enzymes are Fe^2+^, O_2_, and ascorbate while their inhibitors known as oncometabolites include succinate, fumarate, and 2-HG (*R*(−)-2-hydroxyglutarate)

The metabolic and extrametabolic function of AKG in cells and the organism open many different fields for therapeutic interventions for treatment of diseases. Metabolism is closely linked with ageing. The main symptoms of ageing, among others, are disturbances in protein metabolism and altered bone metabolism. Abnormal protein metabolism is also observed after trauma (Engel et al. 2003), surgery (Vinnars et al. 1975), burns (Biolo et al. 2000), or infections (Askanazi et al. 1980). Given its metabolic properties, AKG may be useful in reducing this type of disorders. Moreover, altered metabolism is a feature of cancer cells (Jeong and Haigis 2015). Evidence from recent years has shown that the mitochondrial genes of Krebs cycle enzymes may function as oncogenes or tumour suppressors by influencing different cellular processes. Genes encoding succinate dehydrogenase (SDH) and fumarate hydratase (FH) act as tumour suppressors (Astuti et al. 2001; Baysal et al. 2000; Burnichon et al. 2010; Castro-Vega et al. 2014; Tomlinson et al. 2002). Mutations thereof lead to accumulation of succinate and fumarate, structural analogues of AKG and oncometabolites, which have a tumourigenic role by inhibiting the activity of PHD enzymes and inducing pseudo-hypoxia (Pollard et al. 2005). In contrast, isocitrate dehydrogenase (IDH1/2) genes (products of which catalyse the oxidative decarboxylation of isocitrate to AKG) act as oncogenes (Amary et al. 2011; Borger et al. 2012; Mardis et al. 2009; Parsons et al. 2008). Mutations in these genes lead to production of changed enzymes, which reduce AKG to another oncometabolite—*R*(−)-2-hydroxyglutarate (2HG) (Dang et al. 2009). All the oncometabolites mentioned above modulate (inhibit) the activity of PHD, TET, and KDM enzymes and in this way they participate in the pathogenesis of many cancers. In this case, AKG could be used for reactivation of 2-OGDD enzymes and reversal of metabolic alterations (Fig. 1).

## The Formation of AKG in the Krebs Cycle and its Metabolism

AKG is a key intermediate in the TCA cycle, the energy-producing process that occurs in cells. TCA is a cyclic pathway of eight enzymatic reactions, which oxidises compounds derived from glucose, fatty acids, and amino acids in the matrix of mitochondria, leading to formation of CO_2_ and reduced coenzymes (NADH and FADH_2_). These coenzymes feed electrons to the respiratory chain further used to generate ATP. In the TCA cycle, AKG is formed from isocitrate by oxidative decarboxylation catalysed by IDH. It can be further converted by AKG dehydrogenase to succinyl-CoA and NADH (Krebs and Johnson 1980).

The amount of AKG produced in mitochondria depends on the state of oxidation–reduction (redox). The advantage of NAD^+^ over NADH leads to oxidative decarboxylation of AKG and formation of succinyl-CoA, while in the case of an increased concentration of NADH and a shortage of NAD^+^, reductive transamination of AKG takes place with the participation of glutamate dehydrogenase, leading to formation of glutamate (Owen et al. 2002). Glutamate formed in this reaction can then, in the reaction involving glutamine synthetase, attach another ammonium ion, which results in formation of glutamine (Krebs 1935). AKG can also be produced in the reaction of glutamate and pyruvate catalysed by glutamate pyruvate transaminases (GPT1/2). Additionally, the reversible transfer of an amino group (NH_3_^+^) from glutamate to oxaloacetate also results in the formation of AKG (and aspartate). This reaction is catalysed by glutamate oxaloacetate transaminase, which exists in cytoplasmic and inner-membrane mitochondrial forms, GOT1 and GOT2, respectively (Yudkoff et al. 1994; for review see Sookoian and Pirola 2015).

The TCA cycle metabolites can penetrate to the cytoplasm, where they can be used as precursors for biosynthetic reactions. AKG can freely diffuse through channels (such as voltage-dependent anion channels) in the outer mitochondrial membrane, and it is transported across the inner mitochondrial membrane through the oxoglutarate carrier (OGC), also known as an oxoglutarate/malate antiporter. The OGC plays an important role in the malate-aspartate shuttle and the oxoglutarate-citrate (isocitrate) shuttle (Chappell 1968; for review see Monné et al. 2013; Palmieri et al. 1972).

Because AKG is a key intermediate in the Krebs cycle, it is mainly found in cells (in mitochondria and cytoplasm), but it can also be detected in small quantities (µM) in the bloodstream (Martin et al. 1989; Rocchiccioli et al. 1984; Wagner et al. 2010). However, in people over 40 years of age its level is gradually reduced (to the quantity of ng/ml) (Harrison and Pierzynowski 2008). Presumably, AKG present in blood may derive from the bacterial flora inhabiting the intestine, as different bacteria secrete this metabolite (Otto et al. 2011). Interestingly, physiological increases in AKG levels have been observed in the blood of humans after physical exercise (Brugnara et al. 2012), in the liver of starved pigeons (Kaminsky et al. 1982), and in starved *C. elegans* (Chin et al. 2014).

AKG is an important intermediate in the biosynthesis of amino acids. Cataplerotic reactions that prevent the accumulation of excess AKG in the cell are associated with production of two amino acids: glutamate and glutamine, which are very important for energy metabolism. These two amino acids play a key role in many metabolic pathways and determine proper functioning of organs such as kidney, intestine, liver, as well as pancreatic β cells, neurons, and cells of the immune system (Newsholme et al. 2003).

In various physiological and pathological states, the body may experience an increase in protein catabolism. In these states, glutamine from the muscle and lung tissue is released and becomes available to other organs (such as the intestine or kidneys) and to immune cells. To increase its levels in the body and thus reduce the catabolic response, or even increase protein anabolism, a relevant clinical nutrition therapy (enteral and parenteral) is applied (Stehle et al. 1989). However, the use of glutamine in such a therapy has not found widespread use in medicine, due to the instability of aqueous solutions of this amino acid. Nowadays glutamine supplementation in parenteral nutrition is indicated primarily for patients in critical conditions (Al Balushi et al. 2013; Stein et al. 2009). Similarly, glutamate, from which glutamine can be synthesised de novo, is not routinely used in nutritional therapy because of its relative neurotoxicity and poor permeability across cell membranes (Hermanussen and Tresguerres 2005). In contrast, as an exogenous glutamine precursor that can be used in many deficiency states, AKG has been an object of interest for researchers and clinicians for many years. Many in vivo studies (Cynober et al. 1984, 1990; Dąbek et al. 2005; Filip and Pierzynowski 2008; Junghans et al. 2006; Loï et al. 2005) demonstrate that exogenous AKG administered as a dietary supplement in the form of various salts (ornithine, sodium, calcium) is absorbed by the organism and can be metabolised to glutamine and glutamate as well as to other amino acids (proline, arginine).

Experimental studies performed on animals have shown that ingested AKG, glutamine, and glutamate are absorbed in the upper small intestine and then metabolised in enterocytes. During the first pass metabolism in the intestinal mucosa, up to 95 % of glutamate, 70 % of glutamine, and only 40 % of AKG is degraded to CO_2_ (Junghans et al. 2006). The remaining part of AKG may be used in various anabolic pathways, both in the enterocytes and in peripheral tissues, because up to 20 % of dietary AKG appears in the bloodstream (Filip and Pierzynowski 2008). After absorption, this part of AKG can be used for the synthesis of amino acids such as glutamine or proline. AKG is rapidly removed from the bloodstream, and its half-life is less than 5 min (Cynober et al. 1990; Dąbek et al. 2005). The circulating AKG is passed to the liver and kidney using a sodium–potassium pump (Stoll et al. 1991; Welborn et al. 1998). Cell culture experiments have demonstrated that AKG can be easily passed to fibroblasts by simple diffusion (Aussel et al. 1996). However, the other cell culture research suggests that the ability of AKG to penetrate cells is relatively weak but can be increased by the use of its esters (Koivunen et al. 2007; MacKenzie et al. 2007). AKG is fully metabolised by the body and no excretion of the compound in the pure form with urine or faeces is observed. Studies with the sodium salt of ^14^C-labelled AKG revealed the presence of AKG carbon in several tissues (liver, brain, skin, muscle, bone tissue) already after 3 h of administration of the compound (Filip and Pierzynowski 2008).

## AKG and Protection of Cells Against Oxidative Stress

Free radicals also known as ROS are important mediators in cell damage and cell death processes (Sena and Chandel 2012). They are involved in a variety of pathological conditions, attacking various cellular macromolecules. ROS can cause peroxidation of lipids forming cell membranes, structural and functional changes in proteins, or even damage to nucleic acids. Literature data suggest that in different cases of induced oxidative stress under in vitro or in vivo conditions, AKG, like some other metabolites of the Krebs cycle, has antioxidant properties (Andrae et al. 1985). AKG may participate in non-enzymatic oxidative decarboxylation during the decomposition of hydrogen peroxide, which is conditioned by its chemical structure (Long and Halliwell 2011; Sokołowska et al. 1999; Velvizhi et al. 2002a, b; Yamamoto and Mohanan 2003). It has also been shown that AKG can prevent damage to mitochondrial DNA induced by free radicals in mouse neural cells (Yamamoto and Mohanan 2003). Furthermore, AKG inhibited oxidative stress induced in vivo in rats by administration of ammonium acetate or ethanol (Velvizhi et al. 2002a, b). Additionally, it has been detected that AKG administration positively modulates antioxidant levels in rats during induced hepatocarcinogenesis, restoring antioxidants as well as antioxidative enzyme activity to almost normal levels (Dakshayani et al. 2006). In another in vivo study (Varma and Hegde 2004), AKG had a protective effect against oxidative stress-related cataract formation induced by injection of sodium selenite to rat pups.

One of the most interesting features of AKG is its antagonist activity against cyanogens, i.e., materials containing nitriles from which human organism can generate free cyanide at a level that is toxic to the body. Cyanogens may occur naturally or they can be synthetically manufactured; they can be found in various industrial products, household utensils, or even in certain medications (Bhattacharya et al. 2009). Cyanide (hydrocyanic acid salt) is a rapidly acting neurotoxin, the operation of which is related to, among others, induction of oxidative stress in neurons, formation of ROS, and inhibition of the activity of a number of metalloenzymes including the antioxidant enzyme system (Ardelt et al. 1989, 1994; Muller and Krieglstein 1995; Solomonson 1981). The action of cyanide in the cell leads to impaired mitochondrial activity, as a result of which cellular respiration and energy metabolism is inhibited, which finally causes lactic acidosis and cell death (Bhattacharya et al. 2009; Hariharakrishnan et al. 2009). As has been shown, AKG acts as an antagonist of cyanide poisoning, and its protective effect involves, e.g., binding cyanide by a keto group attached to the carboxylic carbon of AKG, which leads to formation of an intermediate—cyanohydrin (Moore et al. 1986; Norris et al. 1990). Furthermore, AKG prevented cyanide-induced reduction in the level of glutathione (an important antioxidant of the cell) and DNA fragmentation in rat thymocytes cultured in vitro (Bhattacharya et al. 2002). Moreover, AKG is known to have protected the brain and liver of rats from damage caused by cyanide-induced ROS activity, and the addition of sodium thiosulphate increased its protective effect (Tulsawani et al. 2005). It also showed the ability to neutralise the oxidative stress caused by cyanogens (Bhattacharya et al. 2009).

Recent studies have shown that the AKG molecule modulates the activity of antioxidant enzymes and stabilises the oxidation–reduction homeostasis in mice of advanced age to the level observed in young animals (Niemiec et al. 2011).

## AKG as a Complementary Factor in States of Protein Deficiency: Clinical and Animal Studies

As a precursor of glutamine, AKG is a molecule with high potential in the treatment of states with increased protein catabolism, such as recovery after trauma, severe infections and burns, or after surgeries. In a number of independent clinical studies, attempts have been made to use exogenous AKG in alleviating this type of disorders by introducing supplementation mainly with an ornithine salt of alpha-ketoglutarate (OKG), consisting of two molecules of ornithine and one molecule of AKG. It has been shown that such a combination is more efficient than AKG or ornithine alone in restoring glutamine pools in muscles (Cynober et al. 2007). The mechanism of OKG action in the body is still unclear, but it is probably multifactorial. OKG activity is associated with an increased synthesis of glutamine, proline, arginine, polyamines as well as with its capacity to induce secretion of anabolic hormones (insulin, growth hormone) and probably with elevated production of nitric oxide from arginine (Cynober 2004). Many studies (Coudray-Lucas et al. 2000; De Bandt et al. 1998; Donati et al. 1999; Hammarqvist et al. 1989, 1990, 1991; Wernerman et al. 1987) showed that OKG administered orally, enterally, or parenterally improved protein metabolism in patients with chronic or acute protein deficiency. Additionally, OKG administration improved nitrogen balance of the body and decreased protein catabolism by reducing muscle proteolysis in patients with severe burns (Coudray-Lucas et al. 2000; De Bandt et al. 1998; Donati et al. 1999), after surgery (Hammarqvist et al. 1989, 1991; Wernerman et al. 1987), trauma, or acute infections (Coudray-Lucas et al. 2000; De Bandt et al. 1998; Donati et al. 1999; Hammarqvist et al. 1990). In patients with burns, administration of OKG also accelerated wound healing (Coudray-Lucas et al. 2000; De Bandt et al. 1998; Donati et al. 1999). OKG also helped to restore metabolic balance by stimulating the secretion of anabolic hormones—insulin, growth hormone (GH), and insulin-like growth factor (IGF)-1 in patients after trauma (Jeevanandam and Petersen 1999). The introduction of OKG supplementation in malnourished patients of advanced age resulted in a significant improvement in their overall health expressed by increased appetite and improved motor skills; it also shortened the time of recovery after severe illnesses or surgery (Blonde-Cynober et al. 2003; Brocker et al. 1994).

Also, many studies on animals with induced muscle catabolism (Jeevanandam et al. 1996; Le Boucher et al. 1997; Ségaud et al. 2005; Vaubourdolle et al. 1988, 1991) showed that OKG modulated protein metabolism by decreasing urea excretion, increasing protein synthesis in the liver and the intestine, and by inhibiting the degradation of myofibrils, reducing total proteolysis, and loss of glutamine from muscle tissue. In addition, OKG exerted a positive effect on the functioning of the intestinal mucosa and contributed to its recovery after surgery (Czernichow et al. 1997; Dumas et al. 1988; Raul et al. 1995). Studies performed on rats have shown that OKG also improves motor skills in healthy individuals (Moinard et al. 2004). It is also known to protect liver cells against damage and prevent a decrease in the activity of the cytochrome P-450 family produced in liver (Roch-Arveiller et al. 1999). Moreover, recent studies have shown that AKG can alleviate intestinal mucosa injury under inflammatory conditions and enhance protein synthesis in intestinal epithelial cells (Hou et al. 2011; Yao et al. 2012).

### AKG as an Immunomodulatory Agent

Glutamine is a known immunoenhancing nutrient in vivo and a modulator of immune cell growth and function in vitro (Abcouwer 2000; Andrews and Griffiths 2002; Saito et al. 1999; Ziegler and Daignault 2000). It modulates the function of monocytes and neutrophils (by increasing phagocytosis and ROS intermediate production) and macrophages (by enhancing cytokine production) involved in the early, non-specific defence response (Furukawa et al. 1997, 2000; Ogle et al. 1994; Wells et al. 1999). Additionally, glutamine is also a fuel for lymphocyte functions (Ardawi 1988) enhancing their proliferation and production of intracellular ROS and glutathione (Chang et al. 1999a) or cytokines (Chang et al. 1999b; Kew et al. 1999). It has been shown that AKG, as a glutamine homologue, has immune-enhancing properties as well, influencing both the non-specific and the specific immune response, especially in stress situations. Studies on rats with burn injures demonstrated that OKG (ornithine alpha-ketoglutarate) displays immunomodulatory properties, because it counteracted the decrease in superoxide anion (O_2_^−·^) generation in polymorphonuclear leukocytes (PMNs) from these animals (Roch-Arveiller et al. 1996). Moreover, in other in vivo studies, OKG enhanced the intracellular production of hydrogen peroxide (H_2_O_2_) and O_2_^−·^ in PMNs and monocytes isolated from rats with induced catabolism, which confirmed that OKG can improve phagocyte response during stress (Moinard et al. 1999, 2002). Additionally, it has been shown that OKG can enhance macrophage cytotoxicity in stress situations by restoring tumour necrosis factor-α secretion and increasing nitric oxide production in stimulated macrophages (Moinard et al. 1999, 2000). Also, exogenous AKG increased O_2_^−·^ and H_2_O_2_ intracellular production as well as myeloperoxidase activity in PMNs in vitro (Mühling et al. 2010). Furthermore, OKG counteracted thymic involution (which is classically associated with a decreased thymocyte count) in burn injured rats and increased tissue concentrations of glutamine and arginine, two essential nutrients for activated immune cells (Le Boucher et al. 1999). OKG can also stimulate various mechanisms of the immune system to an anti-tumour response. It was observed that OKG administered as a dietary supplement to rats with tumours increased the cytostatic activity of macrophages and the cytotoxic activity of natural killer cells (Robinson et al. 1999).

## The Influence of AKG on Bone Tissue

In recent years, numerous papers (Dobrowolski et al. 2008; Filip et al. 2007; Filip 2007; Harrison et al. 2004; Radzki et al. 2012; Tatara et al. 2005, 2006, 2007) have been published to suggest that AKG may have an anabolic effect on bone tissue. Many in vivo studies (Harrison et al. 2004; Tatara et al. 2006, 2007) have demonstrated that supplementation of AKG or its derivatives during the animal growth has positive effects on the development of skeleton by improving the mechanical properties of skeletal bone. Moreover, other in vivo studies have shown that AKG supplementation prevented the development of osteopenia in female ovariectomized rats (Radzki et al. 2012), in rats after gastrectomy (Dobrowolski et al. 2008) or in model of osteopenia induced by denervation in turkeys (Tatara et al. 2005). In a study of menopausal women, it was also observed that administration of AKG (with Ca) inhibited bone resorption and reduced the effects of osteopenia. In women treated with AKG sodium salt, after 24 weeks of treatment, a significant decrease (about 37 %) in the level of CTX in the bloodstream was observed as well as higher bone density of the lumbar spine in comparison with the control group (receiving only CaCO_3_) (Filip et al. 2007). The results of above studies suggest that AKG not only can inhibit bone resorption, but can also induce reconstruction of bone tissue in the states of osteopenia and osteoporosis.

Although the positive influence of AKG on bone mineral density and strength is well documented in many in vivo studies (Dobrowolski et al. 2008; Filip et al. 2007; Harrison et al. 2004; Radzki et al. 2012; Tatara et al. 2005, 2006, 2007), its mechanism has not been elucidated so far. It is believed that AKG can contribute to an increase in the body pool of amino acids necessary for synthesis of type I collagen (proline and hydroxyproline) and thus have a positive effect on bone quality (Harrison and Pierzynowski 2008; Majamaa et al. 1987; Petersen et al. 2003). Some of the results of studies conducted in vivo in humans and animals (Cynober et al. 1990; Jeevanandam and Petersen 1999; Kristensen et al. 2002; Riedel et al. 1996; Tatara et al. 2005, 2006), where it was observed that OKG or AKG sodium salt led to an increase in the serum levels of proline, may be used as a support of this thesis. It is also suggested that AKG can stimulate production of IGF-1 or GH, which are anabolic hormones that regulate bone modelling and remodelling. IGF-1 increases the efficiency of mature osteoblasts by, among others, stimulating collagen synthesis and inhibiting degradation thereof, while GH stimulates proliferation of the osteoblastic cell line and increases the expression of bone morphogenetic proteins, which stimulate osteoblast differentiation and bone formation (Giustina et al. 2008). Moukarzel et al. (1994) showed that parenteral administration of OKG increased the circulating plasma level of IGF-1, which was confirmed by another study (Jeevanandam and Petersen 1999) carried out on grown-ups who were orally supplemented with OKG and in whose circulating blood the levels of IGF-1 and GH were increased. Furthermore, it was demonstrated (Tatara et al. 2012) that oral administration of AKG in combination with beta-hydroxy-beta-methylbutyrate for pregnant sows increased the serum concentration of GH and IGF-1 in their offspring. However, in the studies of Harrison et al. (2004), in which a group of lambs were supplemented with sodium salt of AKG, no increase in IGF-1 plasma levels was observed, yet there was an increase in their bone mineral density. In turn, other in vivo studies (Harrison et al. 2004; Rosen et al. 1995) have demonstrated that IGF-1 stimulates bone growth and increases their size, but does not affect the mineral density. These findings suggest that the positive effect of AKG on bone tissue is probably not associated with stimulation of IGF-1 and GH production when it is administered alone.

The positive effect of AKG on bone tissue may be due to its important role in the biosynthesis of glutamate and glutamine, as well as a multidirectional impact on the synthesis of type I collagen, the main protein of the bone matrix. AKG can participate in the metabolism of bone collagen by various mechanisms. First of all, this metabolite regulates the activity of enzyme P4H, as mentioned above, a member of the 2-OGDDs family, commonly present in various types of cells. P4H occurs inside the endoplasmic reticulum and controls the synthesis of collagen by hydroxylation of proline to 4-hydroxyproline (Hutton et al. 1966; Kivirikko and Pihlajaniemi 1998), which is necessary to form a triple helix of the collagen molecule. Incomplete hydroxylation of proline in the Gly-X-Y sequence results in incorrect formation of the collagen triple helix. Such defective collagen molecules, contrary to the proper ones, are degraded in the endoplasmic reticulum instead of being secreted into the cytoplasm (Lamandé and Bateman 1999; Myllyharju 2003). AKG can also participate in the biosynthesis of collagen by increasing the pool of proline, an amino acid that is the main component of the collagen molecule. The primary source of proline used for biosynthesis of collagen are the products of endogenous and exogenous collagen degradation (Jackson et al. 1975). Proline may also be formed by conversion of AKG to glutamate, and then to pyrroline-5-carboxylate (P5C), which is converted directly into proline (Smith et al. 1980). However, this pathway is less important in acquiring a pool of proline needed for collagen biosynthesis. It is more important that its precursor, i.e. P5C affects the activity of prolidase, an enzyme responsible for cleavage of dipeptides containing C-terminal proline or hydroxyproline and thereby creating a pool of amino acids required for the biosynthesis of collagen (Myara et al. 1984). Karna et al. (2001) have shown in their study that P5C indeed stimulates the production of collagen through activation of prolidase. Therefore, as a precursor of P5C, AKG can significantly affect the metabolism of proline. The contribution of AKG in the biosynthesis of collagen was confirmed in in vitro studies, in which it stimulated the synthesis of procollagen in human skin fibroblasts by increasing the activity of prolidase (Son et al. 2007). Additionally, in vivo studies (Son et al. 2007) have demonstrated a protective effect of AKG on UVB-irradiated skin, since administration thereof to the skin of mice irradiated with UVB reduced the number of wrinkles and decreased the degree of collagen fibre destruction in the skin.

## AKG as an Anticancer Agent

So far, not many investigations of the anticancer activity of AKG have been carried out (Brière et al. 2005; Hou et al. 2014; MacKenzie et al. 2007; Matsumoto et al. 2006, 2009; Robinson et al. 1999; Rzeski et al. 2012; Tennant et al. 2009). However, the results of those few available papers suggest anti-tumour properties of AKG under both normal and reduced oxygen levels and interference of its activity with various mechanisms.

### The Role of AKG in the Regulation of HIF-1 Activity

Recent research suggests that AKG and its structural analogues (succinate, fumarate) are involved in the regulation of signalling pathways engaged in promotion of carcinogenesis (for review see Raimundo et al. 2011).

PHDs 1-3 which catalyse the hydroxylation of proline residues are very important enzymes belonging to the 2-OGDD group. They are known to regulate the activity of HIFs, which play a major role in carcinogenesis, inducing changes in the metabolism of cancer cells (Bruick and McKnight 2001; Epstein et al. 2001; Hirsilä et al. 2003; Jaakkola et al. 2001; Maxwell et al. 2001). The best characterised enzyme of this group is PHD2 (prolyl hydroxylase domain-containing protein 2), encoded by the gene EGLN1, which is proposed to be the most important in the hypoxic response under normoxia (Appelhoff et al. 2004; Berra et al. 2003; Schofield and Ratcliffe 2005). AKG-dependent dioxygenases can potentially be sensitive to the slightest changes in cellular concentrations of this metabolite. This is related to the fact that the Michaelis constant (*K*_m_) of AKG for many dioxygenases is similar to the physiological concentration of this ketoacid (Clifton et al. 2006; Loenarz and Schofield 2008). In addition, the enzymes responsible for the formation of AKG (IDH-1, -2, -3), and for its further processing (AKG dehydrogenase, glutamate dehydrogenases) are strictly controlled.

During the development of solid tumours, large areas with considerable hypoxia are formed. In response to hypoxia, there is a change in the metabolism of cancer cells, enabling them to survive and adapt to the changed, highly stressful microenvironment. Metabolic changes include, among others, intensified glycolysis, increased glycogen synthesis, and use of glutamine (rather than glucose) as the primary substrate for fatty acid synthesis. This metabolic reprogramming is directed at the transcriptional level by the transcription factor HIF-1, which also induces the expression of genes regulating the process of angiogenesis and cell proliferation (Semenza 2013).

There are two types of HIF (HIF-1 and HIF-2) in mammalian cells. Each of them is a heterodimer consisting of one subunit α, which is O_2_-labile, and one stable subunit β. There are three known isoforms of subunit α: HIF-1α, HIF-2α, and HIF-3α. The first one (HIF-1α) is constitutively expressed in all cells, while the other two isoforms (HIF-2α and HIF-3α) are expressed only in certain tissues (including endothelial, lung, renal, and liver cells, as well as the cells of the myeloid lineage). The HIF-3α subunit functions as a dominant negative regulator of HIF-2α and HIF-1α, because by binding to subunit β, it blocks its binding to DNA (Kaelin and Ratcliffe 2008; Maynard et al. 2005; Wiesener et al. 2003). Under aerobic conditions, proline residues (Pro402 and Pro564) located in the oxygen-dependent degradation domain of HIF-1α are hydroxylated by PHD2, which results in recognition of the subunit by the VHL protein (von Hippel-Lindau tumour suppressor), a part of the ubiquitin ligase complex E3. Then, after poly-ubiquitination, HIF-1α is rapidly degraded in the proteasome (Ivan et al. 2001; Kaelin 2005; Yu et al. 2001). Under the oxygen deficiency conditions or at reduced levels of AKG or Fe^2+^, the hydroxylation reaction involving PHD2 slows down or stops, resulting in HIF-1α accumulation in the cytoplasm (Schofield and Ratcliffe 2005). After the translocation into the cell nucleus, HIF-1α dimerises with a stable HIF-1β subunit and the dimer recognises and binds to hypoxia response elements in the genome (Kaelin 2005).

### The Role of Defects in the Krebs Cycle Enzymes in the Regulation of HIF-1 Activity

As mentioned above, under physiological conditions, HIF-1 is activated by hypoxia. However, this factor may also be activated in normal oxygen access, and activation of the HIF-1-mediated response pathway in such conditions is referred as pseudohypoxia. Stabilisation of the HIF-1α subunit occurs, e.g., in the case of a deletion or inactivating mutation in the gene for the VHL protein, which determines the development of tumours, such as human glioblastoma or sporadic renal cell carcinoma (Latif et al. 1993; Ohh 2006; Pugh and Ratcliffe 2003). Also, mutations in the genes for enzymes involved in the metabolism of AKG, which is a cofactor of PHDs, result in stabilisation of the HIF-1α subunit. These mutations occur mainly in three enzymes of the Krebs cycle: SDH, FH, and IDH.

SDH is a heterotetramer complex localised in mitochondria and composed of subunits A, B, C, and D and the SDH5 factor involved in tetramer mounting (Hao et al. 2009). It has been demonstrated that mutations occurring in the individual components of the complex may predispose to the development of certain types of cancer, mainly paragangliomas (Astuti et al. 2001; Baysal et al. 2000, 2002; Burnichon et al. 2010; Niemann and Muller 2000), pheochromocytomas (Astuti et al. 2001), renal cell carcinoma (Ricketts et al. 2008; Vanharanta et al. 2004), T cell acute leukaemia (Baysal 2007), and gastrointestinal stromal tumours (Italiano et al. 2012; Janeway et al. 2011; Stratakis and Carney 2009). FH, on the other hand, is an active homotetramer occurring in the mitochondria and the cytoplasm, recessive mutations in the gene of which are the cause of development of encephalopathy and early death, while its dominant mutations predispose to cancers such as multiple cutaneous and uterine leiomyomas or hereditary leiomyomatosis and renal cell cancer (Castro-Vega et al. 2014; King et al. 2006; Tomlinson et al. 2002). Defects in the SDH and FH enzymes are the cause of accumulation of Krebs cycle intermediates, i.e., succinate and fumarate (structural analogues of AKG) in cells. These compounds compete with AKG in binding to PHD2, and when bound to the enzyme they inhibit its activity, which in turn contributes to stabilisation of HIF-1α and activation of genes that promote carcinogenesis (Brière et al. 2005; Isaacs et al. 2005; Pollard et al. 2005; Pugh and Ratcliffe 2003; Selak et al. 2005).

Mutations in the genes of IDH1 and IDH2 may also affect the activity of HIF-1 (Zhao et al. 2009). NADP^+^ dependent enzymes, IDH1 and IDH2, are homodimers, wherein IDH1 is present in the cytoplasm and peroxisomes, and IDH2 in cell mitochondria (Corpas et al. 1999). It has been shown that mutations in IDH1 and IDH2 are responsible for the development of certain cancer types, including diffuse and anaplastic gliomas, secondary glioblastomas, specific types of cartilaginous tumours, and acute myeloma leukaemia (for review see Dang et al. 2010; Schaap et al. 2013). The mutated enzymes lose their natural ability to transform isocitrate into AKG, instead acquiring the ability to reduce AKG to 2HG. As a result, the pool of mobile AKG is reduced, and the cell accumulates its structural analogue—2HG (Dang et al. 2009; Schaap et al. 2013). According to some authors’ reports, both a reduction in the amount of AKG and an increase in the amount of 2HG can inhibit the activity of PHD2 and activate HIF-1 (Xu et al. 2011; Zhao et al. 2009). Some contradictory reports suggest in turn that the *R*-enantiomer of 2HG stimulates the activity of EGLN1 (PHD2), EGLN2 (PHD1), and to a lesser extent EGLN3 (PHD3) and thus inhibit the activity of factor HIF-1. In contrast, the *S*-enantiomer of 2HG has the capability of inhibiting PHDs (Koivunen et al. 2012; Losman et al. 2013). However, recent studies (Tarhonskaya et al. 2014) have shown that under in vitro conditions, non-enzymatic oxidation of 2HG to AKG may occur (involving reducing agents such as ascorbic acid or the reduced form of l-glutathione), and the level of AKG formed in this process is sufficient to activate PHD2. The results of these studies may suggest a misinterpretation of the previous results (Koivunen et al. 2012), but further studies are needed to determine accurately the effect of 2HG on the activity of PHD2.

### The Role of AKG and its Structural Analogues in the Regulation of Epigenetic Processes

Chemical modifications of chromatin play an important role in regulating the function of the genome and therefore, in cell physiology. Information provided by epigenetic modifications (changes made without modifying the DNA sequence) plays a considerable part in regulating the processes such as transcription or DNA repair and replication. At the same time, the role of factors regulating epigenetic processes is also very important, because changes in the level of their expression or genomic changes in these factors may contribute to induction or maintenance of a variety of tumours (Dawson and Kouzarides 2012). The basic processes regulating chromatin structure and function are DNA methylation and histone post-translational modification (acetylation, methylation, phosphorylation, ubiquitination, biotinylation, and SUMOylation). Many enzymes, including methyltransferases and demethylases, are involved in these processes (Meng et al. 2015). Recent studies indicate that AKG and its structural analogues—succinate, fumarate, and 2HG can regulate the level of DNA and histone methylation, as the main enzymes conducting demethylation/hydroxylation reactions belong to the family of 2-OGDD. The main demethylases of histones are KDM2-7, which remove methyl groups from almost all known methylation sites in histones and can also catalyse the demethylation of three methylated lysines and arginines (Hoffmann et al. 2012). In turn, the process of oxidative DNA demethylation is conducted by TET1-3 hydroxylases, which hydroxylate 5mC to 5-hmC (Ito et al. 2010; Tahiliani et al. 2009). Several studies (Cervera et al. 2009; Chowdhury et al. 2011; Letouzé et al. 2013; Schaap et al. 2013; Xiao et al. 2012; Xu et al. 2011) have shown that both the Krebs cycle intermediate metabolites, succinate and fumarate, and the *R*(−)2HG act as competitive inhibitors of enzymes KDMs and TETs, and that the inhibitory effect enhances methylation of DNA and histones by methyltransferases, which in turn may increase carcinogenesis. Nevertheless, the effect of these structural analogues of AKG can be reversed by AKG itself.

### Anti-Tumour Activity of Exogenous AKG: In Vitro and In Vivo Studies

One of the very important elements of response to hypoxia and HIF-1 activation is transcription of genes playing a key role in angiogenesis. This process is crucial for the development of solid tumours. The growing tumour tissue needs high amounts of oxygen and nutrients that are supplied by diffusion from the nearby blood vessels in the initial stage of tumour development. As the tumour develops and increases its size, the cells of the nearby blood vessels start running out of oxygen, which activates HIF-1 and initiates the process of neoangiogenesis. HIF-1 in tumour cells activates the transcription of genes for pro-angiogenic factors such as vascular endothelial growth factor (VEGF), PDGF-B (platelet-derived growth factor, type B), hepatocyte growth factor, epidermal growth factor, angiopoietin-2, or placental growth factor (Sacewicz et al. 2009). Matsumoto et al. (2006, 2009) showed that exogenous AKG exhibited anti-tumour activity by reducing the level of the HIF-1α subunit and inhibition of angiogenesis in hypoxic conditions. In their study, AKG inhibited the expression of the HIF-1α subunit and the ability to connect HIF-1 protein subunits, decreased the activity of the *Vegf* gene promoter, and, consequently, inhibited VEGF and erythropoietin production in the Hep3B cell line. Furthermore, AKG inhibited tube formation in an in vitro angiogenesis model. Those anti-angiogenic effects of AKG were confirmed in another in vitro study, carried out using the Lewis lung carcinoma (LLC) cell line (Matsumoto et al. 2009). Additionally, AKG administered alone showed anti-tumour activity and enhanced the activity of a chemotherapeutic agent (5-fluorouracil, 5-FU) in vivo. In the mouse dorsal air sac assay, AKG reduced the amount of newly formed blood vessels caused by administration of the cancer cell line (LLC). Also intraperitoneal administration of AKG alone or its combination with 5-FU to mice with transplanted tumours significantly inhibited tumour growth and angiogenesis in tumour tissue (Matsumoto et al. 2009) which suggests the clinical usefulness of this molecule. In turn, the results of the experiments carried out by Brière et al. (2005) and MacKenzie et al. (2007) suggest a possibility of application of AKG in the treatment of neoplastic diseases in which Krebs cycle enzyme defects cause pseudohypoxia and activation of HIF-1 in normoxia. Brière et al. (2005) have shown that exogenous AKG prevented translocation of HIF-1 into the nucleus of fibroblasts with a mutation in the gene for SDHA (succinate dehydrogenase subunit A). In another study, the MacKenzie et al. (2007) have shown that the competitive inhibition of PHDs by succinate or fumarate may be reversed by increasing the cellular level of AKG. However, native AKG does not easily penetrate into cells; therefore, to increase the effectiveness of its function, tests were carried out using cell-permeating AKG derivatives, i.e. AKG esters with increased hydrophobicity—octyl-AKG and 1-trifluoromethyl benzyl-AKG (converted by cytoplasmic esterases to AKG). These derivatives restored the normal activity of PHDs, thereby decreasing the level of HIF-1α in SDH-deficient cells (MacKenzie et al. 2007). Other in vitro studies (Tennant et al. 2009) have shown that AKG esters (1-trifluoromethyl benzyl-AKG, TaAKG) can restore the activity of PHDs under hypoxic conditions, which has far-reaching effects on tumour cells. Restoration of the PHD activity in such conditions not only resulted in destabilisation of HIF-1α, but also induced functional changes in cells—reversal of the hypoxia-induced increased glycolysis process and cell death as its consequence. In addition, it has been shown that AKG derivatives (TaAKG and ETAKG—5-ethyl,4-1-trifluoromethylbenzyl AKG) may also function well in vivo. TaAKG penetrated several layers of cells in spheroids derived from cell line HCT116 (human colon carcinoma) and destabilised their HIF-1α subunit. Moreover, after oral administration of ETAKG to a mouse xenograft tumour model, increased levels of AKG within the tumour tissues were observed, as well as decreased levels of HIF-1α, and reduced glucose metabolism. The results of the studies mentioned above suggest that AKG in the form of a diester can reactivate PHDs and destabilise HIF-1α in vivo, thereby reducing the expression of genes targeted by this factor (Tennant et al. 2009). However, it has recently been shown that not all types of AKG esters that penetrate the cell membrane have the same activity on HIF-1. In the studies of Hou et al. (2014), membrane permeable ester dimethyl AKG (DAKG), which is the precursor of AKG, temporarily stabilized HIF-1α by inhibition of PHD2. During the long-term impact of DAKG on cells under normoxia conditions, an increase in the level of HIF-1α and expression of its target genes was observed, which indicates that DAKG, in contrast to AKG, promotes the state of pseudohypoxia. The authors speculate that at a high availability of nutrients, DAKG may have been quickly converted to succinate or fumarate, which inhibited the activity of PHD2. On the other hand, the activity of PHD2 might have been inhibited by increasing intracellular levels of ROS in pseudohypoxic conditions induced by DAKG.

Additionally, in vitro studies have shown that exogenous AKG may affect the level of DNA methylation (Letouzé et al. 2013). In contrast to genetic mutations, DNA methylation is a reversible process, which creates a possibility of introduction of new drugs for the treatment of certain tumours (Rodríguez-Paredes and Esteller 2011). Cancer cells are often characterised by a decrease in total DNA methylation and by hypermethylation of promoter CpG islands, which results in transcriptional silencing of tumour suppressor genes. This phenotype, which is characterised by simultaneous multiple gene hypermethylation, occurs, for example, in glioma, in which it is the result of mutations in IDH1/IDH2 genes and the inhibiting activity of 2HG on enzymes belonging to the KDM and TET groups. Also in paraganglioma, mutations in the SDH genes determine such a phenotype (Letouzé et al. 2013). In the studies of Letouzé et al. (2013), SDH-deficient chromaffin cells displayed an increased 5-mC/5-hmC ratio and histone methylation, while the addition of AKG to the culture medium reversed the accumulation of 5mC in vitro and consequently changed this phenotype. These results suggest that exogenous AKG can restore the TETs enzyme activity inhibited by succinate accumulated in cells and restore the cellular normal phenotype.

Given the contradictory results obtained in studies using various esters of AKG, currently, it seems to be safer to use AKG in its native form in cancer therapy. This is supported by the fact that recent studies have shown an antiproliferative effect of AKG on three cancer cell lines: Caco-2, HT-29, and LS-180, representing different stages of the development of colon adenocarcinoma (Rzeski et al. 2012). AKG interfered in the cell cycle of the tumour cells by increasing the expression of cyclin-dependent kinase (CDK) inhibitors p21 Waf/CIP1 and p27 Kip1. Affecting the cell cycle by influencing the proteins involved in its regulation (cyclins) is a very promising feature of AKG as a potential anticancer agent. Each stage of the cell cycle is supported by specific cyclins forming complexes with CDKs, which are involved in phosphorylation of certain proteins. This facilitates maintenance of the cycle, and consequently leads to cell division. The above-mentioned CDK inhibitors subsequently inhibit DNA replication and are responsible for cell cycle arrest resulting in the absence of cell division (Meeran and Katiyar 2008; Xiong et al. 1993). The influence of AKG on the cell cycle of tumour cells is not limited to increasing the expression of the CDK inhibitors. It also decreased the protein level of cyclin D1 and inhibited phosphorylation of the key regulator of the cell cycle, i.e., the Rb protein, which resulted in arresting a large number of cells in the G1 phase, preventing them from entering the division phase (Rzeski et al. 2012).

It should be noted that AKG in combination with 5-hydroxymethylfurfural, prepared in the form of an infusion solution, are currently being investigated for the treatment of patients with non-small-cell lung carcinoma, not responding to any conventional therapy. This combination, named KARAL^®^, is currently in phase II clinical study and shows great promises as cancer treatment (Donnarumma et al. 2013).

## Conclusions

Since the discovery and description of the Krebs cycle, a number of features and functions of one of its main metabolites, i.e. AKG have been identified so far. Yet, many of the AKG actions are still waiting to be discovered. However, current knowledge about this metabolite already ensures its practical application in various fields of human life. Today, AKG is synthesised chemically, but attempts have been made at biotechnological production of this metabolite by various bacteria and yeast (Otto et al. 2011). AKG is of particular importance in industry, where it is used as a building material in the chemical synthesis of heterocycles. It is also a major component of new biodegradable, chemoselective, and mechanically adjustable elastomers with a potential application in biomedicine (Barrett and Yousaf 2008). However, the properties of this molecule also allow use thereof in medicine. AKG is used nowadays as a component of solutions for infusion, formulations used in wound healing, or as a dietary supplement. It is used, for example, during cardiac operations in order to avoid disturbances of blood flow and pressure (Kjellman et al. 1997) or in patients after surgery or trauma to prevent muscle breakdown. Many studies indicate that it may be helpful in the treatment of various diseases and solving health problems, e.g., kidney disorders, bacterial and yeast infections, problems with many organs, such as the intestines, stomach, liver, eyes, and even during cyanide intoxication. However, the efficiency of the therapeutic use, in some cases, is still debated. Broadly described, the positive effect of AKG on bone tissue suggests its potential application in prevention of bone formation disorders, in the treatment of diseases with progressive loss of bone mass, such as osteoporosis, or in improving the body’s bone mass. Nevertheless, the mechanism of its action on bone tissue has not been elucidated yet.

Some studies from recent years have provided a lot of new interesting information about the role of AKG as a molecule regulating cell function, which may allow it to be used in the treatment of cancer. Depending on the type of cancer, AKG could affect tumour cells by reversing their metabolic response to hypoxia or pseudohypoxia. The antiproliferative activity of AKG suggests in turn the possibility of restoring oxidative phosphorylation in tumour cells in place of aerobic glycolysis, which is characteristic to all cancers, in states of increased availability of this metabolite to tumour cells. However, additional studies are required to confirm this hypothesis. Additionally, for the therapeutic efficacy of AKG, it is also important to determine which form of supplementation would be the most applicable—oral administration, infusion, or, e.g., administration of AKG in the form of nanomolecules whose action is aimed specifically at the tumour cells. Not until performing additional in vitro studies and clinical trials could we evaluate the effectiveness of AKG as an anticancer agent then. Nevertheless, even the results of the already existing studies suggest a possibility of using AKG, e.g., in chemoprevention or as a support to anti-cancer therapy.

Recent studies suggest that AKG can also regulate the ageing process of the organism and have an influence on prolonging the lifespan (Chin et al. 2014; Salminen et al. 2014). Research performed in the next few years will probably give us an answer to the question whether to expand the characteristics of AKG with another feature, which is maintaining the longevity.
